# Long-Term Immunological and Virological Outcomes in Children Receiving Highly Active Antiretroviral Therapy at Hawassa University College of Medicine and Health Sciences, Southern Ethiopia

**DOI:** 10.1155/2021/2498025

**Published:** 2021-04-09

**Authors:** Demissie Assegu Fenta, Temesgen Bizuayehu Wube, Metsihet Mohammed Nuru

**Affiliations:** Hawassa University College of Medicine and Health Sciences, School of Medical Laboratory Science, Ethiopia

## Abstract

**Purpose:**

To determine immunological and virological failure and associated factors among children infected with human immunodeficiency virus receiving antiretroviral treatments at Hawassa University Hospital, Southern Ethiopia.

**Methods:**

A hospital-based cross-sectional study was conducted among 273 HIV-infected children from July 1 to December 1, 2019. Data were collected using a structured questionnaire and review of patient records. Blood samples for viral load and CD4 count were collected. Data were analyzed using SPSS version 20. Significance group comparison was done by the Kaplan-Meier log-rank test. The Cox proportional hazard model was used to select significant factors of the variability between groups.

**Results:**

A total of 273 children, between the age ranges of 1 to 14 years, were included. Of these, 139 (50.9%) and 134 (49.1%) were males and females, respectively. Children from the rural area were almost five times more vulnerable for virological and immunological failure than those children from the urban area (AOR = 4.912, (1.276-8.815), *P* = 0.032). The overall viral load suppression was 196 (71.8%) with a good adherence of 226 (82.9%). Nonsuppressed HIV viral load was found to be 77 (28.2%) which had two times more viral load copies (AOR = 2.01, (1.21–2.66), *P* = 0.001) when compared to those who had suppressed viral load copies. The proportions of children who had immunological nonresponse were 45.6% (21 out of 46), 30.4% (14 out of 46), and 23.9% (11 out of 46) among children with baseline CD4 of <200, 201-500, and >500 cells/*μ*l, respectively. Unimproved outcomes among females were noted for immunological and virological failure in this study (AOR = 1.901, (1.038-3.481), *P* = 0.038).

**Conclusion:**

In conclusion, the highly active antiretroviral treatment appeared highly effective in terms of immunological and virological long-term outcomes. However, viral suppression (71.8%) in our study was far apart from the UNAIDS target of 90% in 2020. For that reason, strengthening adherence counseling and early initiation of HAART is important.

## 1. Background

Globally, there were still 38 million people living with HIV, 690,000 AIDS-related deaths, and 1.7 million new infections at the end of 2019 [1]. As a result of rigorous international efforts to respond to HIV, coverage of services has been steadily increasing. In 2019, 68% of adults and 53% of children living with HIV globally were receiving lifelong antiretroviral therapy (ART) [1] By June 2020, 26 million people were accessing antiretroviral therapy, marking a 2.4% increase from an estimate of 25.4 million at the end of 2019 [1].

Among 38 million people living with HIV, about 1.8 million were HIV-infected children; of these, more than 80% live in Sub-Saharan Africa [2] In the absence of any intervention for HIV, up to 52 and 75% of children die before the age of two and five years, respectively [3]. According to the 2017 World Health Organization (WHO) report, Ethiopia is one of the highly affected countries with an estimated 65,000 children living with HIV. The estimated number of AIDS-related deaths of children occurring annually was 3200. The reported number of children receiving antiretroviral therapy (ART) was 21,700 [3, 4].

As early initiation of ART has shown benefits, for this reason, WHO has recommended initiation of ART for all children (<10 years of age) and adolescents (10–19 years) living with HIV, regardless of WHO clinical stage or CD4 cell count [5]. Based on this recommendation, ART coverage in children living with HIV has increased in many countries resulting in a decrease in morbidity and mortality due to HIV infection.

Efficacy of highly active antiretroviral treatment (HAART) is monitored by both clinical and laboratory measures, including estimation of HIV-1 viral load and CD4 cell count, while on treatment. World Health Organization recommends viral load estimation as the preferred monitoring approach to diagnose and confirm treatment failure [6]. However, in low-income countries, such monitoring method has become difficult due to inadequate laboratory facilities, shortage of trained staff, and expensive reagents. The success of ART depends on the maintenance of long-term virological suppression, which is particularly challenging in children living with HIV. Varied response to different first-line regimens has been reported from pediatric observational cohorts from different regions of the world [7]. Despite the reduction in morbidity and mortality, a considerable proportion of patients fail to achieve a sustained virological response to therapy [7].

Acquired immunodeficiency syndrome is a systemic disorder characterized by severe impairment and progressive damage of both cellular and humoral immunological response, hematological abnormalities, and virological failure which are strong predictors of morbidity and mortality in HIV-infected children [8, 9]. HIV replicates not only in CD4 lymphocyte cells to a lesser extent in macrophages and dendritic cells [10, 11] but also such replication is followed by immune system depression, which can lead to life-threatening opportunistic infections.

Immunological failure is defined as a fall of CD4 count to pretherapy baseline, 50% fall from the on-treatment peak value, or persistent CD4 levels below 100 cells/mm^3^ [12]. Knowing factors that can help to predict treatment failure can help to identify children that are at higher risk of treatment failure, to change regimen for those who already have failed regimen, and to delay through maximizing follow-up in those who have a potential to failure [12]. From the multiple factors and variables, the documented factors associated with immunological failure include poor adherence, low baseline CD4 cell count, low baseline hemoglobin value, low baseline weight, initiation in lower-level facilities, nondisclosure of HIV status, HIV/TB coinfection, and having an ambulatory functional status at baseline [13]. Other factors such as WHO clinical stage 3 and 4, longer duration on ART, higher baseline CD4 cell count, history of changing care and treatment clinics (CTC), and lack of treatment supporter [13] can also result in immunological failure during HAART treatment.

A significant number of HIV-infected patients fail to achieve sustained virological suppression and immunological recovery to HAART treatment [14]. Even after 6-12 months of first-line treatment, several children are failing to attain viral suppression and it could be because these children may have been infected with mutant-resistant viruses from mothers that were already on treatment with first-line HAART [15].

Viral load is the gold standard and most important indicator of response for HIV treatment and monitoring that could show the amount of HIV genetic material [16] circulating in the blood plasma of the patient at the initiation of HAART. Prior to ARV exposure, high baseline plasma viral load, certain ART regimen combinations, and primary infection with drug-resistant strains of HIV also pose a serious threat to the sustained success of ART [13].

In 2020, WHO established 90% of all people living with HIV will know their HIV status, 90% of all people with diagnosed HIV infection will receive sustained antiretroviral therapy, and 90% of all people receiving antiretroviral therapy will have viral suppression to have an undetectable HIV viral load (VL) as a global target [17]. Achieving viral suppression protects the body's immune system, helps people living with HIV stay healthy, and prevents transmission of HIV to other people. If a patient on treatment is found to have detectable viral replication, it may be due to poor drug adherence and/or drug resistance [18]. In Ethiopia, the three 90s targets have also been introduced by the Ministry of Health [19].

HIV-infected children are considered a priority group for routine viral load monitoring [20]. A threshold of plasma viral load suppression was classified as <1000 copies/ml as suppressed, and a plasma viral load of >1000 copies/ml was classified as an unsuppressed or defined as a viral failure [19]. If both the viral failure and low level of adherence were well addressed, a switch to a second-line ART drug regimen can be early achieved [21]. Accordingly, regular monitoring and evaluation of viral suppression are very important to achieve the established targets and take necessary corrective actions. However, there is limited data regarding viral suppression rate and immunological response among children receiving HAART in Ethiopia including our setting.

Therefore, this study was aimed at assessing the long-term immunological response and virological failure among children receiving HAART at HUCMHS (Hawassa University College of Medicine and Health Sciences) Pediatrics ART Clinic, Southern Ethiopia.

## 2. Methods

### 2.1. Study Design

A hospital-based cross-sectional study was conducted from July to December 2019 on 273 HIV-infected children receiving HAART.

### 2.2. Study Area

The study was done at Hawassa University Comprehensive Specialized Hospital which is one of the tertiary hospitals in the country with a catchment population of 15-22 million people and one of the largest academic institutions in Ethiopia and located at the heart of Hawassa City, the capital city of the SNNPR Regional State and 275 km from Addis Ababa, the capital city of Ethiopia. The altitude of the city is 1697 meters above sea level with the mean annual temperature and rainfall of 20.9°C and 997.6 mm, respectively. The hospital started delivering ART services in 2005 both for children and adults. Currently, there are 273 HIV-positive children enrolled in chronic follow-up care. The HIV follow-up clinic is scheduled daily, both in the morning and afternoon. The average patient census returning for follow-up was 15–20 patients per day.

### 2.3. Source Population

The source population was all children with confirmed HIV-positive status at Hawassa University College of Medicine and Health Sciences, Southern Ethiopia.

#### 2.3.1. Study Population

HIV-infected children of less than 15 years of age who have been receiving HAART and had a follow-up at Pediatrics ART Clinic at Hawassa University Hospital who have at least two consecutive viral load values and baseline CD4 results were included in the study.

Children on treatment for known immunological and virological failure and receiving transfusion treatment for the last three months of data collection and children with their incomplete information with their lost record and who do not have baseline CD4 count and without a legal guardian or unaccompanied children were excluded from the study.

### 2.4. Sample Size

In this study, 273 HIV-infected children who have baseline viral load and CD4 cell count were included in the study.

### 2.5. Data Collection Tools and Procedures

The sociodemographic and clinical characteristics of the study participants were collected using a pretested structured questionnaire by interview and review of patient records for baseline characteristics of the study subjects before initiation of HAART.

Baseline CD4 T cell and viral load counts were taken from patient registration at enrollment of patients at 0 months to the study and after every six-month interval following HAART initiation up to 4 years of HAART treatment.

The child's anthropometry results were taken from the child's record, and adherence to HAART was assessed by self-reporting by the caregiver, pill count, and on-time hospital attendance by the child or his/her caregiver. Blood was drawn for complete blood count, CD4 percentage, absolute CD4+ T cell counts, and HIV-1 viral load, once every 6 months.

#### 2.5.1. Laboratory Testing

In the specimen collection and processing, 5 ml of blood was drawn from each participant using a Vacutainer tube containing anticoagulant ethylenediaminetetraacetic acid (EDTA) for complete blood count using hematological autoanalyzer Ruby CELL-DYN 3000 USA (Abbott Laboratories Diagnostics Division, USA), and CD4 percentage and absolute CD4+ T cell counts were measured using the FACSCount flow cytometer (Becton Dickinson Biosciences, San Jose, CA) and plasma HIV-RNA levels were determined using Abbott Real-Time HIV-1 assay (Abbott Molecular Inc., Des Plaines, IL) with the lower and higher detection limit of 150 and 10,000,000 RNA copies per ml, respectively.

### 2.6. Quality Assurance

The performance of the analyzers was controlled by running quality control material alongside the study participant's sample. To ensure the quality of data, training was given to data collectors and supervisors. A pretest was done on adult patients. The necessary feedback was offered to data collectors the next morning. The laboratory measurements were done by experienced laboratory technologists. The data collection process was supervised by the investigator. A pretest was done on adult patients.

### 2.7. Data Analysis

The data were cleaned, checked, entered, and analyzed using SPSS version 20 statistical software for analysis. Frequencies were presented to describe study participants. The association between outcome (viral suppression) and independent (age, sex, test reason, treatment combination, and adherence) variables was measured using the backward likelihood ratio (LR) method in the multivariable logistic regressions. The Kaplan-Meier method was used to assess the proportion of patients with virologic and immunologic failure, and Cox proportional hazards regression was used to assess the relative risk of failure of each variable. Variables with a *P* value of ≤0.05 were included in the model and selected as variables to fit a model that best explains the variance in the equation.

#### 2.7.1. Definitions

Clinical failure is defined as the appearance or reappearance of WHO clinical stage 3 or stage 4 events after at least 24 weeks on ART in a treatment-adherent child [22].

Immunological failure and anemia are defined as developing or returning to the following age-related immunological thresholds after at least 24 weeks on ART, in a treatment-adherent child and anemia [22]. CD4 count of <200 cells/*μ*l or percent CD4 cell count of <10% is for a child ≥1 year to <5 years of age, and a CD4 count of <100 cells/*μ*l is for a child 5 years of age or older.

Virological failure is defined as a persistent HIV viral load of ≥1,000 copies/ml, after at least 24 weeks on ART, in a treatment-adherent child [23].

#### 2.7.2. Ethical Consideration

This study was reviewed and approved by the Institutional Review Board (IRB) of Hawassa University College of Medicine and Health Sciences. A support letter was obtained from HUCSH Chief Clinical Director's Office, and a permission was obtained from the Pediatrics ART Clinic to collect the necessary data. An informed written consent was taken from the caretakers and assent was obtained from older children (above 8 years old) before enrollment in the study. Then, the objective of this research was explained to the study participants, and their caretakers and those willing to participate were included. To ensure confidentiality of participant's information, anonymous typing was applied. Each participant was interviewed alone to keep privacy. Test results were given to the clinicians who are working on the Pediatrics ART Clinic of the hospital for further diagnosis and management.

## 3. Results

### 3.1. Sociodemographic and Clinical Characteristics of Participants

A total of 273 HIV-infected children and their medical records at the Pediatrics ART Clinic of Hawassa University Hospital were included and reviewed to assess the sociodemographic and clinical characteristics of the study participants described in Table 1. The mean treatment period for HAART in this study was 26.5 ± 1.4 months (Table 1).

### 3.2. Clinical, Virological, and Immunological Characteristics

A total of 273 HIV-infected children and their medical records were reviewed to assess the baseline and 48-month characteristics of CD4 count (immunological) and virological responses. 26.7% and 57.9% of children were at WHO stage 1 at baseline and 48 months of therapy, respectively. There was a good treatment adherence in 226 (82.9%) participants. Other characteristics of HIV-infected children at baseline and 48 months of HAART treatment were depicted in Table 2.

**Figure 1:**
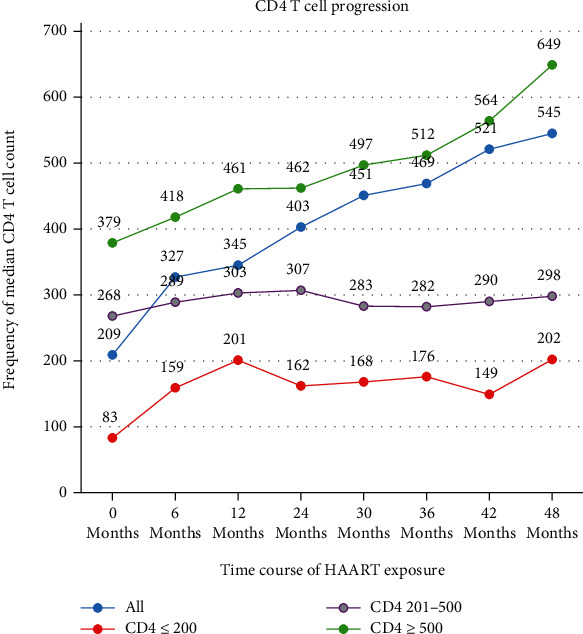
Trends of median CD4 T cell count at baseline and 48 months of HAART treatment.

The immunological response was evaluated and monitored every 6-month interval based on the serial count of CD4+ T lymphocyte (absolute and percentages), measuring of viral load copies, determination of hematological parameters, and clinical data. According to this scenario, the percentage of immunological nonresponse was 16.8% (46 children out of 273 patients). The proportions of patients who had immunological nonresponse were 45.6% (21 out of 46), 30.4% (14 out of 46), and 23.9% (11 out of 46) among children with baseline CD4 of <200, 201-500, and >500 cells/*μ*l, respectively. The median and IQR CD4 cell count at baseline and 48 months of HAART treatment at different CD4 cell levels were 83 (39-172), 268 (203-311), and 379 (362-399) and 197 (185-208), 304 (274-314), and 597 (498-701) cells/*μ*l of CD4 of ≤200, 201-500, and >500 cells/*μ*l, respectively. The trends of the median CD4 T lymphocyte count were indicated below (Figure 1).

Furthermore, immunological progression is indicated by the increasing trend of CD4+ T cell median percentage and IQR of 13.5 (9.3-17.8) at baseline and 21.5 (15.3-30.8) at 48 months of HAART receiving. Similarly, the mean hemoglobin level also increased from 10 mg/dl at baseline to 13.1 mg/dl at 48 months of HAART therapy.

### 3.3. Predictors of Viral Load Suppression and Immunological Failure

Multivariable analysis was used by the Kaplan-Meier method to assess the proportion of patients with virologic and immunologic failure, and Cox proportional hazards regression was used to assess the relative risk of failure of each variable as depicted in Table 3.

## 4. Discussion

In the current study, we assessed the rate of viral load suppression, immunological response failure, and hematological abnormalities among children receiving HAART at Hawassa University ART Clinic for the reason that identification of such problems is essential to make interventions during drug and disease management among children receiving HAART for a prolonged duration of time [24].

Unimproved outcomes among female children were noted for immunological and virological failure in the current study (AOR = 1.901, (1.038-3.481), *P* = 0.038) which was almost 2 times more risky than males for immunological failure. Similar findings were reported from Tigray, Ethiopia [25], Spain [26], and India [27]. Noncomparable findings to our study were reported from Jimma, Ethiopia [28] and Thai HIV-infected children [29]. This difference might be because most of the patients belonged to lower socioeconomic and male-dominated societies, better health-seeking behavior of males than females.

Children being rural residents are statistically significant with immunological and virological failure than urban residents in our study (AOR = 4.912, (1.276-8.815), *P* = 0.032), which is almost 5 times higher risk to develop immunological and virological failure. Inconsistency to this finding was reported from Northeast Ethiopia where being an urban resident was significantly associated with virological and immunological failure than a rural resident [30].

In our study, the overall rate of nonsuppressed HIV viral load was 28.2%; this is an indicator that the suppression rate is still 71.8%, far from the 90% target to be achieved in 2020 by UNAIDS [17]. A higher viral suppression rate was reported from Ethiopia (87%) [31], Jimma, Ethiopia (88.5%) [28], and Zambia (79.5%) [32], but lower than our study finding was reported from Tanzania (25%) [33], from a review of five eastern-southern African countries (62%) [20], and a study from Mali (61%) [34]. In line with our finding was reported from Benin (69.5%) [35].

The proportion of children with undetectable HIV-RNA in our study was 184 (63.4%) which was lower than the finding reported from Thai HIV-infected children (73%) [29] and a study from Oromia, Ethiopia (72%) [36]. In the present study, a total of good adherence for antiretroviral therapy was 226 (82.9%), which was higher than the study reported from Nigeria (80) (65.6%) [37].

The median CD4 cell counts (cells/mm^3^) (IQR) in our study at baseline and 48 months of treatment were 298 (39–453) and 512 (209-1053), respectively. A similar increasing trend was observed from other studies in Ethiopia [31] indicating 345 (17–1435) to 998 (678–2205) cells/mm^3^. A total of 173 (63.3%) children had a CD4 T lymphocyte percentage of ≥25% at 48 months of treatment, 43 (15.8%) between 15-25%, and 57 (20.9%) less than 15% indicating the total immunologic response of 79.1% Almost similar findings were reported from Spain (62.1%) [16]. Lower CD4 T lymphocyte percentage than the current finding was reported from Cameroon (>25%) (46.3%) and (<25%) (53.7%) [38].

In the current study, the risk of developing immunological and virological failure was significantly 2 times higher than children who were at WHO clinical stage 4 (*P* = 0.019) compared to those at stage 1. Findings in line with our report were reported from Benin [35].

In our study, children who had poor adherence were 2 times more likely to develop immunological and virological failure (*P* = 0.021) than those who were in good adherence. A similar finding was reported from the Tanzanian study indicated that poor adherence was significantly associated with (AOR = 15.4, (6.6-36.1), *P* = 0.0001) [39] and in Addis Ababa, Ethiopia (AOR = 0.371, 2.5 (0.3-18.2), *P* = 0.005) [40].

Nevirapine-based regimen was significantly associated with virological and immunological failure compared to those children who were on EFV-based treatment (AOR = 1.90, (1.41-2.56), *P* = 0.001) or which is 2 times higher in virological and immunological failure than EFV-based treatment. Consistent findings were reported from Tanzania (AOR = 4.1, (1.6-10.4), *P* = 0.003) [39], Uganda [41], and Bahir Dar, Ethiopia (AOR = 1.90, 95% CI: 1.41–2.56, *P* = 0.001) [24]. Contrasting finding from our study was reported from Asella, Ethiopia [42] that indicated HAART regimen variability was not significantly associated with recovery of CD4 counts and viral load failure, Addis Ababa, Ethiopia [43] and in Uganda study [44].

In a total of 273 patients enrolled, 43 (15.7%) met the criteria of virological failure. The odds of having virological failure were independently associated with baseline CD4 counts of <200 cells/*μ*l (AOR = 1.328, (1.025-18.9), *P* = 0.012), poor adherence (AOR = 2.051, (1.90–5.45), *P* = 0.021), and nevirapine-based regimen (AOR = 1.90, (1.41-2.56), *P* = 0.003). However, there were no statistical significance associations (*P* > 0.05) between educational status, age group, TB infection, parasitic infection, and other treatments taken during HAART.

## 5. Conclusion

In conclusion, the highly active antiretroviral treatment appeared highly effective in terms of immunological and virological long-term outcomes. However, viral suppression (71.8%) in our study was far apart from the UNAIDS target of 90%. The sustainability of viral suppression and immunological response, once attained, was also variable. Sex, residence, baseline CD4 cell count (≤200 cells/mm^3^), poor adherence, and NVP-based regimen were significantly associated with virological suppression and immunological recovery failure in this study.

Therefore, adherence counseling, follow-up, early initiation of HAART, and switching from one regimen to the other regimen are important for better immune recovery and viral load suppression among HIV-infected children. It is also important for health professionals to make treatment decisions on continuation and treatment change for those receiving HAART for a prolonged period and initiation of HAART for antiretroviral-naïve children. Furthermore, large-scale cohort studies are recommended to determine virological and immunological responses and drug resistance patterns of children who are on a single-drug regimen for a long period to strengthen and explore the problem in-depth.

## Figures and Tables

**Table 1:** Sociodemographic and clinical characteristics of HIV-infected children at Hawassa University Hospital (*N* = 273).

Variables	Category	Frequency (%)
Age in years	≤7	53 (19.4)
	>7	220 (80.6)
Mean age (SD)		10.2 ± 3.2
Sex	Male	139 (50.9)
	Female	134 (49.1)
Residence	Urban	49 (17.9)
	Rural	224 (82.1)
Educational status	Did not begin	38 (13.9)
	Primary	219 (80.2)
	Secondary	16 (5.9)
Family size	≤3	46 (16.8)
	4-7	192 (70.3)
	>7	35 (12.8)
Diarrheal disease	Yes	17 (6.2)
	No	256 (93.8)
Intestinal parasites	Yes	38 (13.9)
	No	235 (86.1)
Malaria	Yes	5 (1.8)
	No	268 (98.2)
TB	Yes	7 (2.6%)
	No	266 (97.4)
URTI and LRTI	Yes	9 (3.3)
	No	264 (96.7)
Skin infection	Yes	3 (1.1)
	No	270 (98.9)
Temperature (°C)	≤37	261 (95.6)
	>37	12 (4.4)
Other medications	No	229 (83.9)
	Cotrimoxazole	31 (11.4)
	Amoxicillin	5 (1.8)
	Augmentin	2 (0.7)
	Nutritional supplement	6 (2.2)

SD: standard deviation; TB: tuberculosis; URTI: upper respiratory tract infection; LRTI: lower respiratory tract infection.

**Table 2:** Immunological, virological, and clinical characteristics of HIV-infected children at baseline and 48 months (*N* = 273).

Variable	Baseline value	At 48 months
Mean age in years (SD)	7.6 (3.1) years	10.2 ± 3.2 years
Median WAZ score (IQR)	-1.3 (-2.4 to -0.07)	-1.2 (−2.7 to −1.5)
Median HAZ score (IQR)	-0.2 (-0.5 to -0.6)	-1.6 (−2.6 to −1.3)
Median WHZ score (IQR)	-1.2 (−1.9 to −0.5)	-1.1 (-2.2 to -0.1)
Median CD4 cell count (cells/mm^3^) (IQR)	298 (39–453)	512 (209-1053)
CD4 cell count (cells/mm^3^)		
≤200	124 (45.4)	15 (5.5)
201-500	132 (48.4)	52 (19.0)
>500	17 (6.2)	206 (75.5)
Mean (SD), Hb (mg/dl)	10 (1.6)	13.1 (1.75)
Anemia		
Yes	111 (40.7)	31 (11.4)
No	162 (59.3)	242 (88.6)
Median CD4+ T lymphocyte (IQR)	16.5 (12.3-20.8)	21.5 (15.3-30.8)
CD4+ T lymphocyte percentage		
>25%	81 (29.7)	173 (63.3)
15-25%	69 (25.2)	43 (15.8)
<15%	123 (45.1)	57 (20.9)
WHO clinical stage		
Stage 1	62 (22.7)	158 (57.9)
Stage 2	94 (34.4)	102 (37.4)
Stage 3	90 (32.96)	8 (2.9)
Stage 4	27 (9.89)	5 (1.8)
Viral load (copies/ml)		
<1000	39 (14.3)	166 (60.8)
5000-10,000	73 (26.7)	60 (21.9)
10,000-100,000	69 (25.3)	29 (10.6)
>100,000	92 (33.7)	18 (6.6)
Adherence		
Good	—	226 (82.9)
Fair	—	29 (10.6)
Poor	—	18 (6.5)
Viral load		
Undetectable	36 (13.2)	184 (68.4)
Detectable	273 (86.8)	89 (32.6)
Viral suppression		
Suppressed	—	196 (71.8)
Not suppressed	—	77 (28.2)
HAART regimen	AZT, 3TC, EFV	37 (13.6)
	AZT 3TC, NVP	124 (45.4)
	D4T, 3TC, EFV	15 (5.5)
	D4T, 3TC, NVP	55 (20.1)
	TDF, 3TC, EFV/NVP	26 (9.5)
	AZT, D4T, 3TC, PI	3 (1.1)
	ABC, 3TC,NVP/EFV/PI	13 (4.8)

ABC: abacavir; AZT: zidovudine; CD: cluster of differentiation; EFV: efavirenz; 3TC: lamivudine; NVP: nevirapine; TDF: tenofovir; PI: protease inhibitor; HAART: highly active antiretroviral treatment; IQR: interquartile range; Hb: hemoglobin; SD: standard deviation; WAZ: weight for age; HAZ: height for age; WHZ: weight for height.

**Table 3:** Factors affecting viral load suppression and the immunological response among HIV-infected children at Hawassa University ART Clinic (*N* = 273).

Variables	AOR (95% CI)	*P* value
Sex		
Male	1	
Female	1.901 (1.038-3.481)	0.038
Age in years		
≤7	1	
>7	0.466 (0.203-1.072)	0.083
Residence		
Rural	4.912 (1.276-8.815)	0.032
Urban	1	
HAART regimen		
AZT, 3TC, EFV	1	
AZT, 3TC, NVP	1.90 (1.41-2.56)	0.001
D4T, 3TC, EFV	1.87 (0.36-9.64)	0.450
D4T, 3TC, NVP	1.76 (1.349-7.9)	0.042
TDF, 3TC, EFV/NVP	1.95 (0.45-2.63)	0.146
AZT, D4T, 3TC, PI	1.90 (0.75-14.81)	0.223
ABC, 3TC, NVP/EFV/PI	1.81 (0.71–4.60)	0.286
WHO clinical stage		
Stage 1	1	
Stage 2	1.025 (0.273-2.814)	0.110
Stage 3	1.094 (0.402-3.165)	0.086
Stage 4	1.987 (1.852-18.128)	0.019
CD4 cell count (cells/mm^3^)		
≤200	1.328 (1.025-18.9)	0.012
201-500	0.532 (0.189-1.244)	0.400
>500	1	
Clinical outcomes		
Weight for age *z*-score	-1.2 (−2.7 to −1.5)	0.015
Height for age *z*-score	-1.6 (−2.6 to −1.3)	0.001
Weight for height *z*-score	-1.1 (-2.2 to -0.1)	0.060
Adherence		
Good	1	
Fair	0.64 (0.32–1.73)	0.251
Poor	2.051 (1.90–5.45)	0.021
Viral load		
Detectable	1	
Undetectable	1.44 (1.08–1.92)	0.015
Viral suppression		
Suppressed	1	
Not suppressed	2.01 (1.21–2.66)	0.001

ABC: abacavir; AOR: adjusted odds ratio; AZT: zidovudine; CD: cluster of differentiation; EFV: efavirenz; 3TC: lamivudine; NVP: nevirapine; TDF: tenofovir; PI: protease inhibitor; HAART: highly active antiretroviral treatment; IQR: interquartile range; Hb: hemoglobin; SD: standard deviation; WAZ: weight for age; HAZ: height for age; WHZ: weight for height.

## Data Availability

The data that support the findings of this study are available from the corresponding author upon reasonable request with the permission of the university and the corresponding author but not publicly available.
